# The risks of warm nights and wet days in the context of climate change: assessing road safety outcomes in Boston, USA and Santo Domingo, Dominican Republic

**DOI:** 10.1186/s40621-021-00342-w

**Published:** 2021-07-19

**Authors:** José Ignacio Nazif-Munoz, Pablo Martínez, Augusta Williams, John Spengler

**Affiliations:** 1 Faculté de médecine et des sciences de la santé, Université de Sherbrooke, 150, place Charles-Le Moyne, Longueuil, QC, J4K 0A8 Canada; 2 Centre de recherche Charles-Le Moyne – Saguenay – Lac-Saint-Jean, 150, place Charles‑Le Moyne, C. P. 200, Longueuil, Canadá; 3 Department of Environmental Health, Harvard T.H. Chan School of Public Health, 401 Park Drive, 4th Floor West, 404N, Boston, MA 02215 USA

**Keywords:** Extreme weather conditions, Road safety, Boston and Santo Domingo, Time-series

## Abstract

**Background:**

There remains a dearth of cross-city comparisons on the impact of climate change through extreme temperature and precipitation events on road safety. We examined trends in traffic fatalities, injuries and property damage associated with high temperatures and heavy rains in Boston (USA) and Santo Domingo (Dominican Republic).

**Methods:**

Official publicly available data on daily traffic outcomes and weather conditions during the warm season (May to September) were used for Boston (2002–2015) and Santo Domingo (2013–2017). Daily maximum temperatures and mean precipitations for each city were considered for classifying *hot days*, *warm days*, and *warm nights*, and *wet*, *very wet*, and *extremely wet days*. Time-series analyses were used to assess the relationship between temperature and precipitation and daily traffic outcomes, using a quasi-Poisson regression.

**Results:**

In Santo Domingo, the presence of a *warm night* increased traffic fatalities with a rate ratio (RR) of 1.31 (95% CI [confidence interval]: 1.00,1.71). In Boston, precipitation factors (particularly, *extremely wet days*) were associated with increments in traffic injuries (RR 1.25, 95% CI: 1.18, 1.32) and property damages (RR 1.42, 95% CI: 1.33, 1.51).

**Conclusion:**

During the warm season, mixed associations between weather conditions and traffic outcomes were found across Santo Domingo and Boston. In Boston, increases in heavy precipitation events were associated with higher traffic injuries and property damage. As climate change-related heavy precipitation events are projected to increase in the USA, the associations found in this study should be of interest for road safety planning in a rapidly changing environment.

**Supplementary Information:**

The online version contains supplementary material available at 10.1186/s40621-021-00342-w.

## Highlights

- This study made cross-city comparisons to analyze trends in road traffic outcomes associated with high temperatures and heavy rains.
- Increasingly heavy precipitation events were strongly and positively associated with traffic-related property damages and nonfatal injuries in the case of Boston.
- Modifying driving behavior through vehicle technology during heavy precipitation events should be paramount to avoid the potential for climate change-related human and economic losses.
- Road safety planning should address climate change-related heavy precipitation events in the USA

## Background

Our understanding of how road safety and climate change interact with each other is becoming utterly important. Road traffic injuries and deaths is a major public health concern. Current global estimates indicate that around 1.2 million people die each year from road crashes, and traffic injuries are the 7th leading cause of disability (Institute for Health Metrics and Evaluation (IHME), 2015). Consequently, road injuries and deaths have been recognized to be serious impediments to sustainable development across countries (Alam & Mahal, 2016; World Health Organization, 2015; Prinja et al., 2015). Simultaneously, global warming has been recognized as one of the major challenges faced by humanity (Theofilatos & Yannis, 2014a). In the coming decades, extreme weather events associated with global warming will test the sustainability, resilience, and flexibility of multiple systems, including road infrastructures and human capabilities (Theofilatos & Yannis, 2014a; Mora et al., 2018). Under these new circumstances knowledge to lure out the multiple effects of climate change on road safety should be advanced. It is thus important to contrast how inclement weather may affect places with different levels of development of road infrastructures, transport policies, vehicles’ technology, and/or health systems.

The association between road traffic collisions and weather has been systematically studied (Mora et al., 2018; Koetse & Rietveld, 2009; Prati et al., 2018; Qiu & Nixon, 2008; Theofilatos & Yannis, 2014b; Razzaghi, 2019; Wang & Zhang, 2017; Saha et al., 2016; Abdel-Aty et al., 2011; Malin et al., 2019; Otte im Kampe E et al., 2016; Parks et al., 2020), particularly with attention to winter conditions (e.g., rain, snow, and extreme cold temperatures). Increasing trends of crashes and nonfatal injuries during rainfall and snowfall have been consistently observed (Mora et al., 2018; Qiu & Nixon, 2008; Abdel-Aty et al., 2011). Notably, rainfall following a dry period has been found to be remarkably dangerous (i.e., significant increment of traffic injuries and property damage) (Qiu & Nixon, 2008). Findings might be explained by detrimental road conditions, malfunction of assistive vehicle technologies, impaired or inexperienced driving, and reduced enforcement under inclement weather, offset by less exposure and more conservative driving behavior during winter season (Mora et al., 2018; Koetse & Rietveld, 2009; Prati et al., 2018; Qiu & Nixon, 2008).

Latest studies have further suggested mixed associations between increases in global temperatures and injury and fatality rates (Malin et al., 2019; Otte im Kampe E et al., 2016). In fact, the current rise in road death rates in the United States might be attributable to warming of the atmosphere (Parks et al., 2020; Robertson, 2018a; Robertson, 2018b). In a recent study (Wu et al., 2018) in continental United States, Wu and colleagues found that traffic fatalities and heat waves were associated with a 3,4% overall increase. However, this effect was not homogenous since fatal crashes in rural areas were positively associated with heat waves by 6%, but no effects were observed for high temperatures and traffic fatalities in urban areas. In another study in Hong Kong a positive but not significant association was found between road injuries under hot weather conditions. More specifically, hot weather was associated with an 11% increase in road injuries (Xing et al., 2019). Studies from other latitudes have pointed in similar directions (Mora et al., 2018; Ambo et al., 2020), however the effect of hot weather on road risk exposure may be parabolic. For instance, while in Australia people tend to cycle more the warmer the weather (Phung & Rose, 2008), in Canada (Miranda-Moreno & Nosal, 2011) and China (Zhan et al., 2020) at very high temperatures, individuals retreat from cycling and walking, thus decreasing exposure to road risks for cyclists and pedestrians. Other studies have considered during hot weather seasons the impact of rainfall on traffic fatalities and injuries. In Texas, Jackson and Sharif (Jacson and Sharif 2016) found no association between rainfall and traffic fatalities, and in Australia effects of these weather conditions were not observed in crash variation (Keay & Simmonds, 2006). On the other hand, in Israel (Brodsky & Hakkert, 1988) and Honk Kong (Xing et al., 2019) significant increases for road injuries were observed by magnitudes of 6 and 19% respectively.

Different pathways from extreme weather conditions to road traffic outcomes have been suggested. For instance, extreme hot weather may deteriorate road users’ performance by increasing tiredness, inattention, or reckless crossing (Abdel-Aty et al., 2011; Böcker et al., 2013; Abedi & Sadeghi-Bazargani, 2017). Additionally, warmer temperatures may be associated with not wearing or using substandard motorcycle personal protective devices (Abdel-Aty et al., 2011; Abedi & Sadeghi-Bazargani, 2017; Malyshkina & Mannering, 2009; Basagaña et al., 2015; Zhai et al., 2019; Craft et al., 2017; de Rome, 2019; Li et al., 2020; Maghsoudi et al., 2018). Further, constant exposure to heat, notably during hot seasons, may adversely affect vehicle conditions by incrementing tire pressure (Abdel-Aty et al., 2011; Parks et al., 2020; Abedi & Sadeghi-Bazargani, 2017). Studies exploring the positive association between rain and road crashes suggest two pathways, loss of friction between tires and roads—making the brake system inefficient, and impaired visibility through either direct rain on the windshield or spray from other vehicles (Jaroszweski & McNamara, 2014).

The impact of climate change through extreme temperature and precipitation events on the transportation system should be further explored to leverage the potential for public policies to prevent human losses due to vehicle collisions. Moreover, as the effects of extreme weather conditions on road traffic injuries and deaths may differ across latitudes, cross-country comparisons can aid to explore such variations across settings. In this study, we thus put our lens on two cities: Boston, MA (United States) and Santo Domingo (Dominican Republic). We have chosen these cities since they represent contrasting cases of road safety policy development. Boston has been selected because safety conditions for cyclists and pedestrians have vastly improved over time (Bongiorno et al., 2019). Boston is the United States’ third most walkable and safest city for pedestrians (National Highway Traffic Safety Administration, 2017). Santo Domingo, on the other hand, cyclists and pedestrians have never been integrated into urban planning schemes (Nazif-Munoz et al., 2020) and as result it has one of the highest traffic fatality rates in the world (Lin, 2016). Ultimately, when road safety systems protect vulnerable road users more consistently, they are more likely to have a stronger road safety system, which protect all road users. We thus examine trends in road traffic fatalities associated with high temperatures and heavy rains using these cities independently, and then finish this work by assessing the extent under which the same weather conditions in Boston affect the number of traffic-crashes with injuries or property damage.

## Methods

Daily meteorological conditions were available from the National Weather Service, for Boston (National Weather Service, 2020), and from the *Oficina Nacional de Metereologí*a, for Santo Domingo (Oficina Nacional de Meteorología (ONAMET), 2020). To reduce seasonal confounding, all analyses were confined to the warm season (May – September). In addition to the continuous value of daily maximum temperature (TMAX), minimum temperature (TMIN), and precipitation, binary variables were created at common thresholds. As Smith et al. (Smith et al., 2013) and Anderson et al. (Anderson & Bell, 2011) concluded the operationalization of hot days affects the relationship between heat exposure and health. As such we opted for two metrics, hot days and warm days. As demonstrated by Kent at al. (Kent et al., 2014) Anderson et al. (Anderson & Bell, 2011) these two measures could be good predictors for mortality. More specifically hot days were defined when TMAX ≥95th percentile of the TMAX for each city, and warm days when 85th percentile ≤ TMAX ≤95th percentile. We also use warm nights when TMIN ≥95th percentile to explore the extent under which traffic outcomes were related to this condition. Indeed, crash response maybe different for daytime and nighttime (Keay & Simmonds, 2006). For precipitation, wet days were considered days with any precipitation, very wet days were those with precipitation exceeding the warm-season daily mean precipitation, and extremely wet days were those with precipitation exceeding the daily mean precipitation of wet days.

Data on daily traffic fatalities and injuries that occurred in Boston, Massachusetts, were available from the Massachusetts Department of Transportation Interactive Mapping Portal for Analysis and Crash Tracking (IMPACT) for 2002–2015 (Department of Transportation Interactive Mapping Portal for Analysis and Crash Tracking, n.d.). Traffic fatalities for Santo Domingo were available from the Dominican Republic Ministry of Public Health for 2013–2017 (Ministerio de Salud Pública, Republica Dominicana, 2018). No personal data were used in this study and therefore ethics approval was not required.

Relative risk (RR) analyses (Williams et al., 2020) were used to determine each city’s relative risk of traffic outcomes on a hot or wet day compared with days below thresholds set a priori. The RR quasi-Poisson regression model equation (Eq. 1) is as follows:

1 $$ \mathit{\log}\ \left({\mu}_j\right)={\beta}_0+{\beta}_1{I}_j\left[ HotDay\right] $$

Where *μ*_*j*_ is the expected number of traffic outcomes for each city on day *j*, and *I*_*j*_[*HotDay*] is a binary indicator of a hot day. The same model was run substituting the indicator for hot day with the other temperature and precipitation indicators. To assess differences across RRs, we also calculated the ratio of relative risks (RRR) (Altman & Bland, 2003).

Time-series (TS) analyses (Imai et al., 2015) were used to assess the relationship between temperature and precipitation and daily traffic fatalities per unit increase in temperature, using a quasi-Poisson regression. We use TS because it allows us to effectively account for time-varying factors such as temperature or precipitations in a series of traffic outcomes by simultaneously introducing functions of time to avoid any potential confounding produced by either season or long-term trends in the series (Imai et al., 2015). For this, we restricted the warm-season TS models using a non-parametric spline with 1 degree of freedom (df) per season to account for any long-term trends in traffic outcomes. To account for unobserved trends, a natural cubic spline with 2 df for the continuous temperature or precipitation variable, determined a priori based on previous research (Wang, 1998), and to optimize the Generalized Cross Validation (GCV) criteria, was added. Day of the week was controlled for with indicator variables for each day, with reference to Friday. The TS model equation (Eq. 2) is as follows:

2 $$ \mathit{\log}\left({\mu}_j\right)={\beta}_0+s\left({\beta}_{1j}\left({T}_{MAX}\right)\right)+{\beta}_2{I}_j\left[ HotDay\right]+{\beta}_3{I}_j\left( Day\  of\ Week\right)+s\left({\beta}_{4j}(Date)\right) $$

where *μ*_*j*_ is the expected number of traffic outcome for each city on day *j*, *I*_*j*_[*HotDay*] is a binary indicator of a hot day, *I*_*j*_[*Day of Week*] is an indicator for day of week, *s* represents splines on variables with non-linear trends. The same model was run substituting temperature variables for precipitation variables and for each city of interest.

## Results

### Fatal collisions in Boston, MA

There was a daily average of 0.07 traffic fatalities per day during the warm season in Boston, MA from 2002 to 2015, and an average of 1.5 traffic fatalities per year 100,000 population, which corresponds to a total of 146 traffic fatalities during this period (Table 1). When evaluating the RRs of traffic fatalities in Boston, MA, there were no precipitation or temperature factors significantly associated with fatalities during the warm season (Table 2). An interesting association however is between traffic fatalities and warm night with an RR = 1.08, (95% confidence interval [CI]: 0.97, 1.20).

**Table 1:** Descriptive statistics of traffic fatalities, meteorological conditions for Boston, MA (2002–2015) during the warm season. p indicates percentile

	Boston, MA
Total Fatalities	146
Daily Mean Fatalities	0.07
Daily Max Temperature [°C]	24.33
Daily Min Temperature [°C]	15.69
Total Hot Days (T_MAX_ ≥ 95thp)	115
Total Warm Days (85^th^p ≤ T_MAX_ ≤ 95^th^p)	300
Total Warm Nights (T_MIN_ ≤ 95^th^p)	119
Mean Daily Precipitation (all days) [mm]	3.05
Mean Daily Precipitation (days with any rain) [mm]	9.04
Total Wet Days (> 0 mm)	707
Total Very Wet Days (>mean)	419
Total Extremely Wet Days (>mean of rainy days)	236
Population	685,094

**Table 2:** Relative risk (RR) with 95% confidence interval (CI) of traffic fatalities on warm-season days with extreme heat or precipitation for Boston, MA

Condition	Boston, MA	
	RR	95% CI
Hot Days (T_MAX_ 95^th^p)	0.95	(0.84, 1.06)
Warm Days (85^th^p ≤ T_MAX_ 95^th^p)	1.09	(0.90, 1.13)
Warm Nights (T_MIN_ ≤ 95^th^p)	1.08	(0.97, 1.20)
Wet Days (> 0 mm)	1.01	(0.93, 1.09)
Very Wet Days (>mean)	0.99	(0.90, 1.09)
Extremely Wet Days (>mean of rainy days)	0.95	(0.83, 1.08)

p indicates percentile

### Fatal collisions in Santo Domingo, RD

In Santo Domingo, there were 1.53 traffic fatalities per day on average during the warm season, and an average of 14.1 traffic fatalities per year 100,000 population, which corresponds to a total of 1404 fatalities from 2013 to 2017 (Table 3). Similar to Boston the presence of a warm night (TMIN ≤95th percentile) preceding fatal traffic incidents was associated with an RR = 1.31 (95% CI: 1.00, 1.71) (Table 4), and the other variables did not capture significant variation for this outcome.

**Table 3:** Descriptive statistics of traffic fatalities, meteorological conditions for Santo Domingo, DR (2013–2017) during the warm season. p indicates percentile

	Santo Domingo, DR
Total Fatalities	1404
Daily Mean Fatalities	1.53
Daily Max Temperature [°C]	32.24
Daily Min Temperature [°C]	24.83
Total Hot Days (T_MAX_ ≥ 95^th^p)	66
Total Warm Days (85^th^p ≤ T_MAX_ ≤ 95^th^p)	104
Total Warm Nights (T_MIN_ ≤ 95^th^p)	55
Mean Daily Precipitation (all days) [mm]	5.56
Mean Daily Precipitation (days with any rain) [mm]	12.21
Total Wet Days (> 0 mm)	481
Total Very Wet Days (>mean)	302
Total Extremely Wet Days (>mean of rainy days)	218
Population	3,318,000

**Table 4:** Relative risk (RR) with 95% confidence interval (CI) of traffic fatalities on warm-season days with extreme heat or precipitation for Santo Domingo, DR

Condition	Santo Domingo, DR	
	RR	95% CI
Hot Days (T_MAX_ 95^th^p)	1.08	(0.82, 1.41)
Warm Days (85^th^p ≤ T_MAX_ 95^th^p)	1.09	(0.87, 1.35)
Warm Nights (T_MIN_ ≤ 95^th^p)	1.31	(1.00, 1.71)
Wet Days (> 0 mm)	0.88	(0.76, 1.03)
Very Wet Days (>mean)	0.95	(0.79, 1.15)
Extremely Wet Days (>mean of rainy days)	1.06	(0.84, 1.32)

p indicates percentile

### Time series analyses of Boston, MA USA and Santo Domingo, RD

The estimated effects of maximum temperature at nights were nonlinear for traffic fatalities in both cities. In Boston, higher RR of traffic fatalities at hot temperatures are observed. However at average temperatures, traffic fatalities are stable, and then decline at lower temperatures (Figure S1 in Supplementary Material). In Santo Domingo, on the other hand, the response curve suggests increases of RRs in traffic fatalities at average temperatures, whereas extreme temperatures tend to have wide confidence intervals and lower RRs (Figure S2 in Supplementary Material).

### Non-fatal injury and property damage collisions in Boston, MA, USA

For Boston, data were also available on traffic collisions that resulted in non-fatal injuries and property damage. During this study period, there were 25,275 collisions resulting in non-fatal injury and 40,936 in property damage. When analyzing these data, we observe four groups of findings (Table 5). First, except for hot days and traffic injuries in which a non-significant increase of 9% (RR: 1.09 95% CI: 0.99, 1.21) was observed, warm days do not capture concomitant variation in non-fatal injuries nor property damage. Second, warm nights suggest decreases by 13% (RR: 0.87 95% CI: 0.78, 0.98) in non-fatal injury rates, and by 9% (RR: 0.91 95% CI: 0.82, 1.01) in property damage rates. Third, all the precipitation thresholds considered resulted in significantly greater RR of non-fatal injury collisions and property damage. Further, increases in wet conditions are associated with higher RRs of property damage. For instance, the RR of collisions resulting in property damage on wet days is 1.25 (95% CI: 1.19, 1.31), whereas for this outcome on extremely wet days the RR is 1.42 (95% CI: 1.33, 1.51). The ratio of RR (RRR) for these two RRs is 1.05 (95% CI: 1.02, 1.10), suggesting significant higher risks of collisions resulting in property damage on extremely wet days relative to wet days.

**Table 5:** Relative risk (RR) with 95% confidence interval (CI) of traffic collisions resulting in non-fatal injury or property damage on warm-season days with extreme heat or precipitation in Boston, MA

Condition	Non-Fatal Injury	Property Damage		
	RR	95% CI	RR	95% CI
Hot Days (T_MAX_ ≥ 95^th^p)	1.09	(0.99, 1.21)	0.96	(0.86, 1.07)
Warm Days (85^th^p ≤ T_MAX_ ≤ 95^th^p)	0.98	(0.91, 1.05)	0.94	(0.88, 1.01)
Warm Nights (T_MIN_ ≤ 95^th^p)	**0.87**	**(0.78, 0.98)**	0.91	(0.82, 1.01)
Wet Days (> 0 mm)	**1.19**	**(1.13, 1.25)**	**1.25**	**(1.19, 1.31)**
Very Wet Days (>mean)	**1.27**	**(1.19, 1.34)**	**1.36**	**(1.29, 1.43)**
Extremely Wet Days (>mean of rainy days)	**1.25**	**(1.16, 1.34)**	**1.42**	**(1.33, 1.51)**

Bold indicates RRs for which the 95% CI exclude the null (RR = 1.0), and p indicates percentile

Lastly, when comparing the risks of collisions resulting in non-fatal injuries (RR: 1.25 95% CI: 1.16, 1.34) and property damages (RR: 1.42 95% CI: 1.33, 1.51) with the risk for traffic fatalities (RR: 0.95 95% CI: 0.83, 1.08) on extremely wet days, we obtain the following RRRs: for non-fatal injuries 1.12 (95% CI:1.06, 1.19) and for property damage 1.19 (95% CI:1.11, 1.27). These comparisons suggest that on very wet days, non-fatal injuries and property damage have a 12 and 19% higher association with weather conditions than does traffic fatalities.

## Discussion

Understanding how traffic crashes are distributed in consideration to extreme weather events related to climate change requires the development of studies which contrast dissimilar territories (Bergel-Hayat et al., 2013). For this, our study analyzed Boston and Santo Domingo independently. While there were some interesting signals between variables, we did not find however that overall extreme weather conditions associated with heat or rain were consistently capturing concomitant variation on traffic fatality rates in both cities. Nevertheless, when analyses were extended to road injuries and property damages in Boston, our findings suggest a series of complex associations. While variables operationalizing high temperatures at different times signaled mild decreases in non-fatal injuries and property damages, different thresholds to wet days, however, strongly indicated increases in these two outcomes.

To understand findings related to fatal and non-fatal crashes, we need to identify both different road risks’ (temperatures, rain, number of trips) exposure levels (frequency or intensity), and second, a combination of these road risks (Hakkert et al., 2002). First, lack of variation in the fatal outcomes is consistent with previous studies which have stratified analyses by rural and urban settings (Xing et al., 2019). While Wu et al. found that traffic fatalities increased by 3.4% at the presence of heat waves in the United States, in their study this association was null for urban settings. In our study, while the null statistical significance of the effects across Boston and Santo Domingo was similar, the sign and magnitude of these results, however, prevents us from suggesting the presence of unique pathways between high temperatures and traffic fatalities in highly contrasting cities. We can only suggest for Boston that increases in temperatures may be associated with decreasing in the number of road trips as property damage in Boston may have decreased—albeit not significantly. In short, if very high temperatures are present, road users in this city may avoid ground travelling, which in turn reduces the chances of experiencing road crashes and fatal ones (Otte im Kampe E et al., 2016). However, this null finding may also indicate that if high temperatures do not necessarily affect the number of trips, drivers may be mitigating its adverse effects on driving performance (i.e., increase of tiredness, or inattention (Abdel-Aty et al., 2011; Xing et al., 2019; Böcker et al., 2013; Abedi & Sadeghi-Bazargani, 2017)) by using vehicles’ air conditioners or opening vehicles’ windows. For instance, the mitigating effects of air conditioners in households on negative health outcomes has been documented in United States and China (Ostro et al., 2010; Ma et al., 2014). While part of this explanation could be applied to Boston since studies have suggested significant increases in vehicles’ air conditioners during warm seasons (Gately et al., 2017), it is also important to acknowledge that people living in warmer areas, as Santo Domingo, tend to adapt to hot temperatures (Lian et al., 2020; Bobb et al., 2014), and therefore effects of high temperatures on driving performance may not be as acute. When health and fire services attend to collisions on time, the probability of traffic deaths decreases (Sánchez-Mangas et al., 2010). Since in Boston increasing of high temperatures has been associated with raises in police, health services and fire services dispatches (Williams et al., 2020), then it is possible that Boston’s emergency medical services have provided prompt attention to severe car crashes to the point of successfully decreasing traffic fatalities. This interpretation is reinforced with our result of hot days associated with increases in injuries by 9% (95% CI: − 1.0, 21.0) (which is consistent with a non-significant increase also found in Hong Kong of 11% (95% CI: − 4.0, 30.0) in this association). While high temperatures may deteriorate road users’ performance by increasing tiredness or inattention, which in turn leads to an augmentation of road injuries, the timely attention from medical services may be indeed reducing the risk of fatalities for these crash victims. Last, another element derived from these results is the analogous association between warm nights and traffic fatalities. In our study this association was positive and non-significant for both cities albeit much higher for Santo Domingo with a 31% (95% CI: 0.0, 71.0) increase. In Wu et al.’s study this association was also non-significant and positive with a 7.1% increase (95% CI: − 1.2-16.1) which is highly similar to our results from Boston, where we observed an 8.0% increase (95% CI: − 3.0, 20.0). Further, studies have suggested that this association can be explained by the prevalence of risk driving behavior such as driving under the influence of alcohol which is correlated with this type of weather condition (Xing et al., 2019). In the Dominican Republic, various studies have indicated that alcohol consumption increases the risk of traffic injuries by more than 3 times at the national level (Shinar, 2012, Cherpitel et al. 2021) and by more than 4 times in Santo Domingo (Borges et al., 2017).

Second, the presence of wet days being associated with higher increases of road injuries and property damage relative to traffic fatalities, as our findings of RRRs indicate, suggests three interrelated hypotheses. First, other studies have noticed that increments in rains are linked with falls in the severity of crashes (from fatal, to injury, to property damage) (Doherty et al., 1993; Eisenberg, 2004). Indeed, Edwards (Edwards, 1999) proposed that under inclement wet weather conditions drivers tend to significantly reduce vehicles’ speed, which in turn decreases the impact of a collision but not necessarily the proportion of collisions. A similar finding was also identified by Wu et al. (Wu et al., 2018) in which road traffic fatalities were consistently decreasing the higher the precipitations were observed in continental United States. Our results however are consistent with those of Xing et al. (Xing et al., 2019) in which road injuries are analyzed. In Hong Kong, an increase of 34.0% (95% CI: 16.0–55.0) in road injuries were observed after the presence of heavy precipitation, in our study in Boston, our variable extreme wet days was associated with an increase of 19.0% (95% CI: 16.0–34.0) in this outcome. Complementary to this hypothesis, the increase of road injuries and property damage can also be explained by how vehicles’ brake systems become inefficient under slippery roads. Indeed, tires lose traction to roads when the presence of water mixed with oil is prevalent (Eisenberg, 2004). Last, water also impairs drivers’ visibility through either direct rain on the windshield or spray from other vehicles (Jaroszweski & McNamara, 2014).

The pattern found for Boston, where increases in precipitation were associated with higher traffic injuries and property damage, are indeed of particular interest for road safety planning in the USA. The Fourth National Climate Assessment (NCA4) (U.S. Global Change Research Program, 2018), completed in November 2018, stated that heavy precipitation events linked to climate change are expected to increase during the twenty-first century, particularly affecting Northeastern United States. Thus, as extremely wet days will become more common, their incidence on number of traffic injuries and property damage should be more carefully considered.

Our evaluation of the general association of extreme weather conditions during warm seasons has the following limitations. First, our exposure variable is measuring daily averages of temperatures and precipitations. Variation in these two variables could be however better captured by having access to hourly information. This would allow us to identify a more precise correspondence between extreme weather conditions and the time when collisions occurred. Nevertheless, since we were limited to daily information for our exposure variables, we could only use day as unit of analysis. Second, traffic outcomes were not broken-down according to drivers, cyclists, pedestrians, sex, or age groups. Recent studies have suggested that males relatively to females are more likely to be affected by heat waves as well as individuals aged 46–65 are also more likely to be affected by these weather conditions (Xing et al., 2019). It is possible thus that our results are masking important differences between these demographics. In terms of road users, effects of temperature increases may be, relative to motor vehicle drivers, more detrimental in cyclists. Third, as it is common in this literature, the examination of enforcement variables remains an important challenge. The effects of the weather could indeed be mediated by the type and quantity of emergency services responses during these conditions. Fourth, our estimates may be confounded by other unmeasured time-varying variables such as decreases in helmet use in Santo Domingo or increases in safety motor vehicle requirements in Boston. In this regard it is important to highlight that while our initial effort was to attempt to find similarities to understand pathways between two highly contrasting cities, cross-city comparisons should adjust for other time varying factors linked to road safety capacity more precisely. Last, and relatedly to our fourth limitation, to have more adequate cross-city comparisons analysis should consider matching periods of analysis. In our case Boston considered the 2002–2015 period whereas Santo Domingo a shorter one with 2013–2017.

## Conclusions

What type of policies and follow up studies could be explored from these findings and their related hypotheses? An appropriate intervention would be to test weather drivers, cyclist and pedestrian behaviors could be modified during adverse weather conditions through devices such as vehicle’s dashboards, mobile phone alerts and portable road signs. For this, studies on mobile applications and vehicle’s dashboards should be designed considering how the sudden provision of warning information must necessarily offset driver and pedestrian distraction. Analyses of portable road signs should for instance consider which type of messages in form and content are effective to immediately reduce speed. Depending on how traffic rules are designed and enforced in specific administrative units, immediate and provisional legal reduction of speed limits under extreme wet weather, and road quality maintenance and improvement with attention to mitigation of slippery conditions should also be studied. Regarding the presence of hot temperatures, studies should consider more carefully the use of air conditioners and how these indeed contribute to road safety. It is important to acknowledge however that excessive and malfunctioning use of air conditioners in vehicles has been associated with increment of external and car cabins air pollution (Gately et al., 2017; Gołofit-Szymczak et al., 2019). Second, analyses of temperature effects on road outcomes with attention to emergency dispatches should be explicitly carried out. This would provide a better understanding of how potential overcrowding of emergency systems during extreme weather conditions maybe or not affecting medical responses.

## Supplementary Information

**Additional file 1:**

## Data Availability

Not applicable.
