# Supplementary figures and images for: The risks of warm nights and wet days in the context of climate change: assessing road safety outcomes in Boston, USA and Santo Domingo, Dominican Republic
Source: Inj Epidemiol. 2021 Jul 19;8:47. doi: 10.1186/s40621-021-00342-w (PMC8287725; doi:10.1186/s40621-021-00342-w)

**Supplementary material**


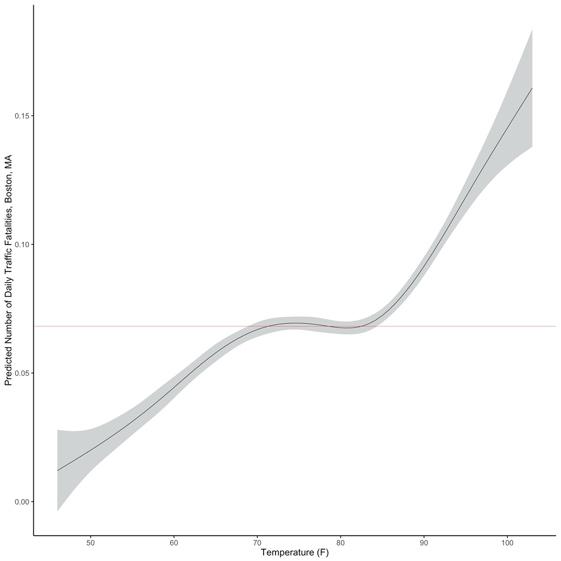
Figure S1 – Boston

Figure S2 – Santo Domingo


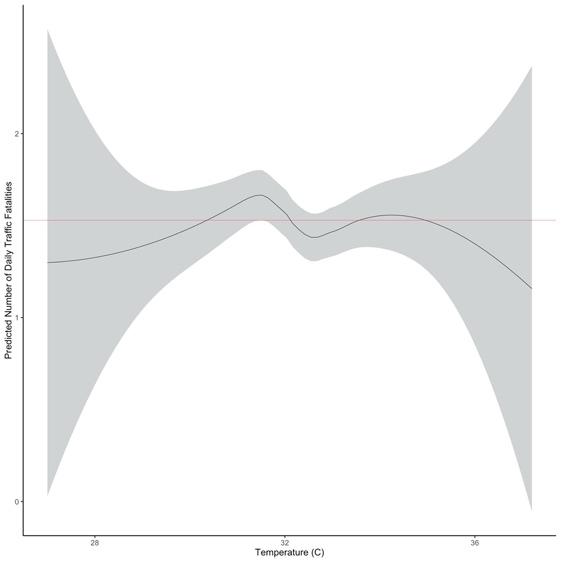

Supplement: Supplementary file 1 — Additional file 1: [file 40621_2021_342_MOESM1_ESM.docx]
